# Targeted mapping of quantitative trait locus regions for rhizomatousness in chromosome SBI-01 and analysis of overwintering in a *Sorghum bicolor* × *S. propinquum* population

**DOI:** 10.1007/s11032-012-9778-8

**Published:** 2012-09-06

**Authors:** Jacob D. Washburn, Seth C. Murray, Byron L. Burson, Robert R. Klein, Russell W. Jessup

**Affiliations:** 1 Department of Soil and Crop Sciences, Texas A&M University, 370 Olsen Blvd 2474 TAMU, College Station, TX 77843-2474 USA; 2 Crop Germplasm Research Unit, USDA-ARS, 2881 F&B Road, College Station, TX 77845 USA

**Keywords:** Perennialism, Perenniality, Rhizomes, *Sorghum*, *Sorghum propinquum*

## Abstract

While rhizome formation is intimately associated with perennialism and the derived benefit of sustainability, the introduction of this trait into temperate-zone adapted *Sorghum* cultivars requires precise knowledge of the genetics conditioning this trait in order to minimize the risk of weediness (e.g., Johnsongrass, *S. halepense*) while maximizing the productivity of perennial sorghum. As an incremental step towards dissecting the genetics of perennialism, a segregating F4 heterogeneous inbred family derived from a cross between *S. bicolor* and *S. propinquum* was phenotyped in both field and greenhouse environments for traits related to over-wintering and rhizome formation. An unseasonably cold winter in 2011 provided high selection pressure, and hence 74.8 % of the population did not survive. This severe selection pressure for cold tolerance allowed the resolution of two previously unidentified over-wintering quantitative trait locus (QTL) and more powerful correlation models than previously reported. Conflicting with previous reports, a maximum of 33 % of over-wintering variation could be explained by above-ground shoot formation from rhizomes; however, every over-wintering plant exhibited rhizome growth. Thus, while rhizome formation is required for over-wintering, other factors also determine survival in this interspecific population. The fine mapping of a previously reported rhizome QTL on sorghum chromosome SBI-01 was conducted by targeting this genomic region with additional simple sequence repeat markers. Fine mapping reduced the 2-LOD rhizome QTL interval from ~59 to ~14.5 Mb, which represents a 75 % reduction in physical distance and a 53 % reduction in the number of putative genes in the locus.

**Electronic supplementary material:**

The online version of this article (doi:10.1007/s11032-012-9778-8) contains supplementary material, which is available to authorized users.

## Introduction

Expanding interest in lignocellulosic biofuels has largely centered on perennial species, although high biomass annuals including photoperiod-sensitive sorghums are receiving considerable attention. One ecosystem-derived benefit of perennial crops is their ability to use less fertilizer and a reduced carbon footprint associated with the over-wintering storage and remobilization of nutrients the following year (Jessup 2009). In addition, perennial cropping systems have the advantage of reduced soil erosion and are able to retain greater water reserves in the soil profile than annual cropping systems (Piper and Kulakow 1994; Cox et al. 2002). Perennials take advantage of earlier and later seasonal growth than annual crops, which theoretically allows for a longer duration of solar energy capture. For these and other reasons, perennial systems have potential economic and environmental benefits capable of facilitating the increased use of marginal soils for crop production (Cox et al. 2002).

The genus *Sorghum* is a desirable genetic model for investigating and utilizing perennialism because it consists of inter-fertile species that behave as both annuals and perennials in cold climates. Prior reports of perennialism in the genus *Sorghum* have largely focused on rhizome inheritance, a trait that is correlated and genetically linked to over-winter survival in sorghum (Ramaswamy 1973; Paterson et al. 1995; Yim and Bayer 1997; Jang et al. 2006; Jang et al. 2009). Rhizomes are modified underground stems that act as carbohydrate storage organs (Whitmire 2011) for many perennial plants. *Sorghum bicolor* (L.) Moench is a tropical, diploid species (2*n* = 2*x* = 20) widely believed to lack rhizomes. It is grown as an annual crop in temperate environments, although rhizomes have been reported in at least one wild accession of *S. bicolor* (L.) Moench subsp. *verticilliflorum* (Steud.) de Wet ex Wiersema & J. Dahlb. (Bhatti et al. 1960; Mouftah and Smith 1969). In contrast, *Sorghum halepense* (L.) Pers. (2*n* = 4*x* = 40) is a weedy sorghum species, commonly known as Johnsongrass, with the ability to persist as a perennial in very cold climates (Warwick et al. 1986). Temperate-climate perennialism in *S. halapense* is correlated with rhizomatousness (Warwick and Black 1983; Warwick et al. 1984, 1986), and similar correlations have been found in other temperate-adapted perennial sorghum species (Paterson et al. 1995).

*Sorghum propinquum* (Kunth) Hitchc. (2*n* = 2*x* = 20) is a wild rhizomatous perennial sorghum found in Asia and the Pacific islands (Magoon 1961). *Sorghum propinquum*, like *S. bicolor*, has only 20 chromosomes; however, its behavior during meiosis is dissimilar to *S. bicolor* in that the nucleolus is organized on a different chromosome (Magoon 1961). *Sorghum propinquum* is cross-compatible with *S. bicolor*, whereas *S. halepense* and *S. bicolor* are very difficult to inter-mate (De Wet 1978). This fact thus makes *S. propinquum* a highly desirable source for the transfer of perennialism genes into cultivated sorghums.

In general, *Sorghum* rhizomes appear to be a critical factor influencing over-wintering, and the ability of rhizomes to survive seems to be due to the insulating effect of the soil (Warwick et al. 1986). The carbohydrate resources within a rhizome that over-winters are generally depleted during the following season, suggesting that rhizomes are temporary storage organs. In fact, many rhizomes disintegrate within 19–24 days after shoots emerge (McWhorter 1961; Monaghan 1979). Another indication that rhizomes are primarily a winter storage organ is that their fresh weights double within 4 days after flowering under ideal conditions (McWhorter 1961; Monaghan 1979).

Both classical and molecular genetics studies have examined rhizome formation in several grass genera, including *Digitaria*, *Oryza*, *Zea*, and *Sorghum* (Hacker 1983; Tao et al. 2003; Sacks et al. 2003; Westerbergh and Doebley 2004; Paterson et al. 1995). In the genus *Digitaria*, it was concluded that rhizomatousness was controlled by two or more incompletely recessive genes (Hacker 1983). Rhizomatous and non-rhizomatous species in the genus *Oryza* have also been studied with varying results (Tao et al. 2003; Sacks et al. 2003). Tao et al. (2003) indicated that rhizomatousness is most likely controlled quantitatively by several quantitative trait loci (QTL) acting in an additive manner. Analysis of rhizome production and perennialism in wide crosses within the genus *Zea* also support a quantitative inheritance pattern (Westerbergh and Doebley 2004).

The genetics of sorghum rhizomes has been investigated more than most other species because of the interest in controlling weediness of Johnsongrass as well as in developing temperately-adapted perennial sorghum applications (Paterson et al. 1995). Classical genetic studies have shown that crossing the rhizomatous species *S. halepense* with a non-rhizomatous *S. bicolor* results in a 3:1 segregation ratio among the F2 progeny, possibly suggesting one major dominant gene controls rhizome formation (Yim and Bayer 1997). Hybrids between *S. sudanense* (Piper) Stapf. and *S. halepense* also exhibit a 3:1 segregation pattern, supporting the one dominant gene hypothesis (Ramaswamy 1973; Yim and Bayer 1997). However, additional studies have reported that rhizome formation is controlled by three dominant, additive genes (Monaghan 1979).

Using a diploid model, Paterson et al. (1995) concluded that rhizome formation in sorghum was a polygenic-inherited trait, as at least nine chromosomal regions associated with quantitative variation in rhizome traits were detected. In rice, the *Rhz2* and *Rhz3* genes co-localize with many of the sorghum loci for rhizomes, indicating that the rhizome production mechanisms may be evolutionarily conserved between these two genera (Hu et al. 2003). In this study, we sought to further investigate the relationship between rhizomatousness and over-wintering in sorghum and the longevity of rhizomatous sorghum lines based on years of re-growth. In addition, we sought to confirm previously identified QTL for rhizome growth and over-wintering and increase the resolution of the region through targeted mapping.

## Materials and methods

### Field cultivation

On May 24, 2010, 130 F3:4 lines derived from hybrids between *S.* *bicolor* and *S.* *propinquum* (Paterson et al. 1995) were planted at College Station, TX, USA (30°32′N, 96°26′W) in a randomized complete block design. Standard sorghum agronomic practices were followed throughout the season with supplemental irrigation, fertilization, and pesticide applications made as necessary.

### Greenhouse cultivation

A separate greenhouse study was also undertaken using the same plant materials, in order to corroborate the field study. Seeds of the F4 lines were planted on November 11–12, 2011, in three replicates in 10-cm^2^ pots filled with Sun Gro^®^ Redi-earth^®^ Plug and Seedling Mix (Sun Gro Horticulture, Vancouver, British Columbia, Canada) and placed in a randomized complete block design. Plants were purposefully grown in small pots under the assumption that limited space would stress the plants and induce early maturation and therefore early rhizome development. Temperatures in the greenhouse were moderated, but fluctuated between 16 and 27 °C. After 3 weeks of growth, seedlings were thinned to 4–6 plants per pot and allowed to continue growth.

### Phenotyping

On November 29, 2010, near the time of the first frost, phenotypic measurements were taken on the field plots to determine the rhizomatousness of each line. These measurements were a modification of those used by Paterson et al. (1995). Measurements included: (1) the number of living plants per line prior to first frost; (2) the average number of rhizome-derived shoots (RDS) originating from each plant; and (3) the average distance of these shoots from the center of the row. Plants remained in the field during the winter and were scored phenotypically for re-growth from rhizomes the following spring (March 31, 2011). Over-wintering ability was defined as the presence of post-winter active re-growth within a plot from the crown or rhizomes, not seedlings. The surviving plants were allowed to grow throughout the summer of 2011 and over-winter in the field for a second season during the winter of 2011–2012 in order to obtain data on the longevity of these lines. In the spring of 2012, the plants were again rated phenotypically for over-wintering and rhizome parameters. Spring phenotyping involved a slight variation from fall measurements in that distance was measured from the center of the crown of each plant rather than the center of the row. Special care was taken to ensure that any seedling-derived plants were uprooted at an early stage so they would not be confused with rhizome-derived re-growth.

On April 10–11, 2012, greenhouse plants were removed from their pots and rated visually for the presence and abundance of rhizomes. Each pot was given a score from 1 to 10 based on the amount of rhizome growth. No rhizome growth was represented by a score of 1 and the maximum amount of rhizome growth was represented by a score of 10.

### Statistical modeling for over-wintering ability

Several statistical models were created to test the hypothesis that over-wintering can be predicted based on rhizome growth. Three linear regressions, a logistical ANOVA, and a logistical regression model were tested. We used different combinations of RDS number per plot and RDS distance from the center of the row to create the models. Statistical models were created and tested using JMP^®^ Software, Version 9 (JMP 2011).

### DNA extraction

Leaf tissue was harvested from 10 to 20 plants of each line (except those with fewer surviving plants) and bulked by line. This tissue was frozen at −80 °C, and subsequently lyophilized for 3 days. A 6-mm hole punch was used to remove several discs of tissue from each lyophilized leaf sample, equaling about 0.17 g of dry tissue, and this was ground using stainless steel beads in a Talboys High Throughput Homogenizer (Troemner, Thorofare, NJ, USA). DNA was then extracted following a normal CTAB procedure (Doyle and Doyle 1987) and quantified using a Nanodrop 1000 (Thermo Fisher Scientific, Wilmington, DE, USA). Working samples of 50 μg/ml were then created by dilution with double deionized water. PCR was performed as previously described (Whitmire 2011; Jessup et al. 2012). The DNA fragments were separated using gel electrophoresis and scored as co-dominant markers following a published scoring procedure (Rodríguez et al. 2001; Whitmire 2011). For electrophoresis, a MEGA-GEL (C.B.S. Scientific, Del Mar, CA, USA) high-throughput vertical unit was used. Gels had a final concentration of 6 % acrylamide, 0.5× TBE (Tris–borate–EDTA) buffer, 0.07 % ammonium persulfate, and 0.08 % TEMED (tetramethylethylenediamine) (Wang et al. 2003; Whitmire 2011; Dowling 2011).

### QTL and data analysis

Sixteen *S. bicolor* genomic simple sequence repeat (SSR) markers mapping to a previously-reported rhizome QTL on sorghum chromosome SBI-01 were selected from a set of 329 SSRs (Whitmire 2011) and used for linkage analysis along with other selected markers (Table 1). According to the published *S. bicolor* genome sequence, these markers are distributed over a 15.71-Mb region within chromosome SBI-01 (Gramene 2011). The SSR markers were used for locus refinement employing all 130 F3:4 lines described above. Marker and trait data were entered into QTL iciMapping (ICIM) version 3.1 software (Wang et al. 2012) for linkage analysis and QTL detection. For linkage analysis, marker data were entered into ICIM as a recombinant inbred line and grouped by a LOD score of 3.00 and a distance of 37.20 cM. Ordering was performed using the SER algorithm within the software package and then rippling with the LogL algorithm. After the ordering was complete, two of the markers were manually ordered to match the published *S. bicolor* genome (Gramene 2011). The QTL data was analyzed with an F3 population structure using biparental populations (BIP) mode. The ICIM-ADD (composite interval mapping) method was used with a step size of 0.5 cM and a probability in stepwise regression of 0.001. Deletion was selected as the means for dealing with missing phenotypic data and the LOD threshold was chosen through 1,000 permutations with a type I error level of 0.05. Single marker analysis (SMA) was also performed using the same number of permutations and error level.

**Table 1:** SSR markers used in final QTL analysis with marker names, forward and reverse primer sequences, and location of marker on SBI-01

Name	Primer sequence	Location
SB062A	attaagggcagcatccaatc	53,605,463
	gattggcaagcgtgtgatac	
SBL1B5I	tggcttctctcctctcctgt	54,856,700
	ggatcacagcttcttgggtt	
SBL1B5H	caaggcgaacaagacatttg	54,856,700
	gtctctgccgtgaaaacaga	
SB0050A	agctagtgtgcataatgggc	58,089,588
	ggtgtcagtctttgacccct	
SBL1B6C	tactatgcgtttgggcgtag	61,357,000
	aaccgccaccgtagttaaag	
SBL1B2F	ataccaaagcggaggagcta	62,191,006
	catgtgaggaccgagtcaac	
SBL1B6G	agatcgaccgttaggtggac	68,002,200
	atgatgctacctcgagtccc	
pSB0088	cgcgcagaatcagaaaaagt	69,318,196
	catccaagttggcgtcttct	

Trait data was transformed prior to analysis in order to achieve normality of the residuals. All field data was transformed using the natural log (ln) function except for RDS count for 2011 which was transformed using an ln(*n* + 1) function (Paterson et al. 1995). Graphical representations of the linkage map and QTL presented in this paper were created using MapChart version 2.2 software (Voorrips 2002).

## Results and discussion

### Phenotyping and over-wintering

We sought to re-examine the relationship between over-wintering and rhizome formation in progeny from interspecific hybrids between cultivated sorghum and *S. propinquum*, while also quantifying the survival of plants over multiple winters. During the first winter season (2010–2011) in College Station, plants were exposed to a minimum temperature of −7 °C and 22 days of below-freezing temperatures (OCS 2012). As a consequence of the extreme temperatures for this locale (Fig. S1), only 25.2 % of the plots of F3:4 lines had surviving plants after the winter and only 8.2 % of the total plants within the family survived. By comparison, the winter of 2011–2012 was much milder, with a minimum temperature of −3 °C and freezing temperatures experienced on only 8 days (OCS 2012). Of the 33 plots of F3:4 lines that survived the first winter, 82 % survived the second winter as well (26 of 33 plots).

The extreme temperatures and the associated high mortality rate (74.8 %) during the first winter allowed powerful discrimination of over-wintering potential, much greater than that seen in previous studies utilizing germplasm from these interspecific hybrids (Paterson et al. 1995). Although drought conditions and wildlife pressure probably caused some confounding effects during the second winter, we saw no evidence of either stress during the first winter, leading to the conclusion that the majority of selection pressure on our population was attributable to the cold temperatures. These harsh winter conditions, therefore, allowed for greater differentiation between cold-tolerant and intolerant plants for future breeding projects and/or new mapping populations, and provide a better understanding of the relationship between over-wintering and rhizome growth.

All of our over-wintering statistical models (Table 2) were significant at the α = 0.01 level but had varying degrees of predictive power in *R* ^2^ coefficients. The logistical model was the most useful, indicating that 33 % of the variation in over-wintering can be explained by the rhizome parameters measured. Contrary to conventional opinion, 67 % of the variation in over-wintering was unaccounted for by rhizomes under the best of the models. This unexplained variation may appear to indicate that rhizome growth is not as important to over-wintering as previously thought. However, there are alternative explanations for the low amount of variation explained by rhizomes in our study. One obvious explanation is that our measurements may not have accounted for all rhizome growth due to the nondestructive measurements that were taken. Rhizome phenotyping was based entirely on above-ground indicators of rhizomes since determining the longevity of plant survival was an important aspect of this study. This means that our method was unable to detect rhizomes that had not produced above-ground shoots. Rhizome depth, for example, has been implicated to play a major role in winter survival because of soil insulation (Warwick et al. 1986). Rhizome depth measurements were not taken during this study because of their invasive nature which would have compromised the plants’ ability to over-winter undisturbed. It should be noted, however, that excavation of plants at the end of the study confirmed the presence of rhizomes, even for the most weakly rhizomatous by above-ground determination. Within our population every surviving plant had rhizomes, indicating that although not the only factor, rhizomes appear necessary for winter survival under our study conditions.

**Table 2:** Parameter estimates from over-wintering regression models for *S. bicolor* × *S. propinquum* F4 hybrids

LogRDS only model	*R*² = 0.25			
Term	Estimate	SE	*t*-ratio	Prob > |*t*|
Intercept	0.11	0.04	2.64	0.0094*
LogRDS	0.25	0.04	6.39	<0.0001*

LogAvgDist only model	*R*² = 0.14			
Term	Estimate	SE	*t*-ratio	Prob > |*t*|
Intercept	−0.1	0.09	−1.03	0.3032
LogAvgDist	0.28	0.06	4.42	<0.0001*

Whole model	*R*² = 0.3			
Term	Estimate	SE	*t*-ratio	Prob > |*t*|
Intercept	−0.13	0.08	−1.58	0.1168
LogRDS	0.25	0.05	5.23	<0.0001*
LogAvgDist	0.18	0.06	2.9	0.0045*

Logistic regression model	*R*² = 0.33			
Term	Estimate	SE	χ^2^	Prob > |*t*|
Intercept	3.94	0.81	23.66	<0.0001*
LogRDS	−2.06	0.55	14.13	0.0002*
LogAvgDist	−0.81	0.53	2.37	0.1239
(LogRDS-0.70) × (LogAvgDist–1.31)	−2.06	0.87	5.6	0.0180*

*SE* standard error

* Significant at alpha <0.05

In addition to rhizomes, other factors likely condition over-wintering, including anti-freeze proteins (Cansev et al. 2009; Kosová et al. 2011), carbohydrate fractions (Whitmire 2011), and cold tolerance of root-ball crown (Livingston and Henson 1998; Livingston et al. 1989). Because all above-ground plant material died during the 2010–2011 winter, and most died during the milder 2011–2012 winter, we conclude that if anti-freeze proteins such as dehydrins or others persisted in our material, they must be present and/or effective only in the below-ground plant structures. Cold resistance within the crown seems unlikely because most RDS in our study originated from areas other than the crown of the plant. Root-ball cold tolerance as a critical factor in over-wintering cannot be ruled out because the rhizomes and root-ball material that we observed were generally closely associated with each other.

### QTL analysis

**Fig. 1:**
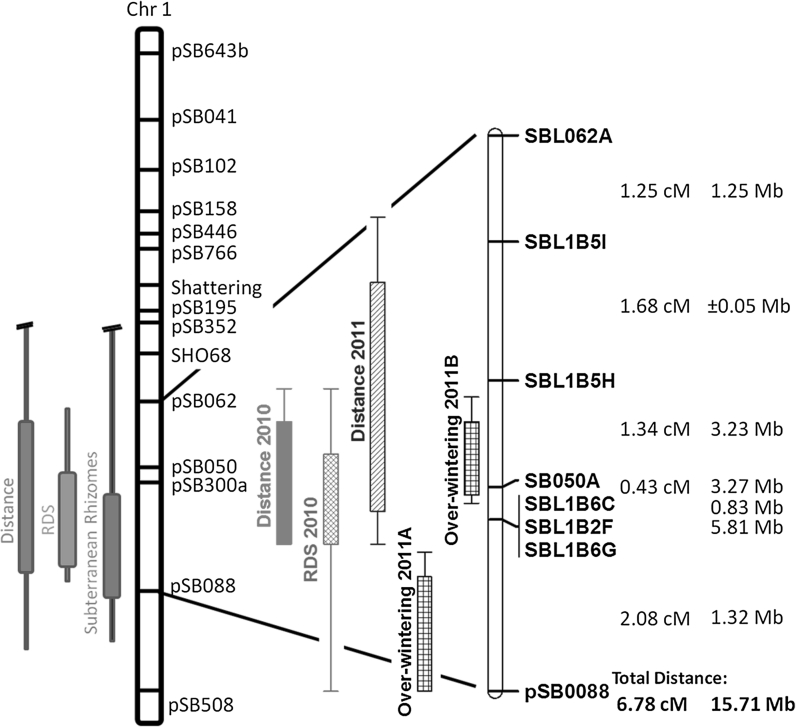
Chromosome 1 QTL map. On the *left*-*hand side* is the full map of sorghum chromosome one (linkage group C) including QTL as reported by Paterson et al. (1995). The remainder of the figure is an enlargement of a section of chromosome 1 between markers pSB062 and pSB0088 (the chosen region of analysis for this study). Calculated map distances in centimorgans along with actual physical distances from the *S. bicolor* genome are displayed next to the figure. QTL marked “Over-wintering 2011A” and “Over-wintering 2011B” come from the same data collected in March 2011

An additional objective of this study was to re-examine a region of sorghum SBI-01 that was previously shown to encode for rhizome formation in interspecific hybrids between *S. bicolor* and *S. propinquum* (Paterson et al. 1995). The completed genome sequence of the parental line BTx623 (Paterson et al. 2009; Goodstein et al. 2012) facilitated the rapid identification of additional molecular markers for refining trait loci. Of 16 new SSRs assayed, six were informative (polymorphic in population, easily scorable). The markers spanned a region with a total linkage distance of 6.78 cM and a total physical distance of 15.71 Mb (Fig. 1). Utilizing these new markers, two novel QTL for over-wintering were detected within the region (Tables 3, 4). To our knowledge, this is the first time over-wintering QTL have been identified in this area of sorghum SBI-01, and we speculate that this likely relates to the over-wintering selection pressure experienced during the first winter. Of great interest to us is the QTL (labeled Over-wintering 2011B) that overlaps directly with a genomic region associated with rhizome growth. At present, we cannot conclude that the same gene or genes in this genomic region are conditioning both over-wintering and rhizome formation, but the co-localization of over-wintering and rhizome formation QTL provide further circumstantial evidence to support the hypothesis that rhizomatousness is an important, perhaps necessary, factor in sorghum’s ability to over-winter.

**Table 3:** Significant QTL regions for *S. bicolor* × *S. propinquum* F4 hybrids: their LOD scores, phenotypic variation explained, additive effect, and dominance effect

Trait name	Position(cM)	Left marker from peak	Right marker from peak	LOD	PVE (%)	Additive effect	Dominance effect
Ln2010Dist	4	SBL1B5H	SB050A	2.81	10.72	−0.09	0.49
Ln2010RDS	4.5	SB050A	SBL1B6C	2.99	14.18	−0.73	0.09
Ln2011Dist	3.5	SBL1B5H	SB050A	2.63	38.25	−0.57	0.57
Over-wintering2011B	4.1	SBL1B5H	SB050A	3.91	11.53	0.00	0.45
Over-wintering2011A	6.1	SBL1B6G	pSB0088	7.09	25.32	−0.03	0.64

**Table 4:** Significant markers from single marker analysis for *S. bicolor* × *S. propinquum* F4 hybrids: their LOD scores, phenotypic variation explained, additive effect, and dominance effect

Trait name	Marker name	Position (cM)	LOD	PVE (%)	Additive effect	Dominance effect
Ln2010Dist	SBL1B5H	2.96	2.43	9.05	−0.21	0.24
Ln2010Dist	SB050A	4.30	4.80	17.07	−0.30	0.42
Ln2010Dist	pSB0088	6.84	2.41	8.98	0.11	0.61
Ln2010RDS	SB050A	4.30	2.19	7.75	−0.70	–0.31
Ln2011Dist	SBL1B5H	2.96	2.38	28.96	−0.62	0.35
Over-wintering 2011	SBL1B5H	2.96	5.95	19.14	−0.05	0.45
Over-wintering 2011	SB050A	4.30	5.67	18.33	−0.16	0.40
Over-wintering 2011	pSB0088	6.84	6.87	21.74	−0.02	0.58

The second region associated with over-wintering on SBI-01 is distinct from the over-wintering QTL discussed above, indicating that two unique genes on SBI-01 are involved in over-wintering. In addition, using the 1-LOD intervals for all QTL, this second region would be considered distinct from any of the rhizome growth-related regions identified in this study. If in fact the causal gene(s) for this region are distinct from those of the rhizome QTL, it is possible that this region represents the contribution of an additional physiological/morphological factor besides rhizome growth that controls sorghum’s over-wintering ability. However, it must be stated that using the 2-LOD regions for the QTL would indicate that this QTL may represent the same causal gene(s) as one of the other identified rhizome QTL in the study, RDS 2010. Additional studies using a larger population size and additional environments and markers are likely required to clarify this observation.

In addition to over-wintering, significant QTL regions were also identified for RDS distance in 2010, RDS count in 2010, and RDS distance in 2011 (Tables 3 and 4 and Fig. 1). These QTL were a confirmation of the original rhizome QTL found by Paterson et al. (1995), but the locus was refined by further marker analysis. Using the 2-LOD intervals from the 2010 data alone, we have narrowed the original QTL region for the rhizome traits measured from a genomic region spanning ~58.7 Mb to a region of ~14.5 Mb. This reduction in the physical size of the locus (75 % reduction) also reduced the number of candidate genes within the QTL region from approximately 2,970–1,407, a 53 % reduction in candidate genes (Paterson et al. 2009; Goodstein et al. 2012). While these results are clearly not adequate to allow identification of candidate genes, the results do indicate that narrowing the locus is readily achievable, given additional markers and larger population sizes. With the advent of whole genome marker analysis utilizing next generation sequencing, the ability to fine map this and other over-wintering loci will not be restricted by the ability to saturate the genome with informative markers. Rather, the ability to accurately phenotype rhizome growth and over-wintering has become the limiting factor in determining genome regions conditioning these important traits.

Our ability to accurately phenotype rhizomes in large populations may make it possible to map QTL for RDS growth. Both our results and those reported by Paterson et al. (1995) show 1-LOD intervals for RDS growth to be smaller than those for RDS distance. Assuming that both of these phenotypes are controlled by the same gene(s), we could conclude that RDS distance is a more variable, and thus less accurate, means of measuring rhizome growth than is RDS count. However, using the 2-LOD regions (in our data only), the opposite trend occurs, with RDS being much larger than RDS distance. Separating the data by year, the 2011 QTL are also appreciably larger than the same QTL from the 2010 data. There are several differences between the phenotyped populations for the 2 years that may explain this discrepancy. One explanation is that all 130 plots, some with rhizomes and some without, were included in the analysis for 2010 while only those that survived the following winter were included for 2011. This means that every plant in the 2011 data had at least some rhizome growth. Also, the 2011 data was collected for only 33 plots. Because the 2010 data included lines that did not survive the winter and lines that had little or no rhizome growth, it may have more power for predicting the regions associated with the presence or absence of rhizome growth. The larger population size of the 2010 data would necessarily give it more statistical power than the data from 2011, which would allow it to identify a smaller interval with more confidence.

To examine rhizome development under controlled environmental conditions, the interspecific sorghum population was planted and grown in greenhouse conditions. It became apparent that the extent of rhizome formation is repressed under these artificial growth conditions. From the greenhouse study, we also discovered that although only limited rhizome development occurred within 10-cm^2^ pots (which is very little space for 4–6 sorghum plants), some development did occur. In fact many of the plants had small rhizome buds which, although difficult to phenotype for size because they were all so similar, were easily distinguished from root growth for counting. Because of the limited rhizome growth, perhaps due to the small pot size, the greenhouse study precluded the detection of significance for rhizome growth on SIB-01. The limited rhizome growth was, however, associated with small broad peaks that overlapped and extended beyond the corresponding regions of the significant QTL from the field environments. In future studies, we anticipate examining parameters (e.g., larger pots, the use of field soil for growth, use of climate-control chambers) that would permit greater rhizome development and hence more accurate phenotyping of QTL conditioning rhizome formation. In addition, optimizing greenhouse conditions for rhizome formation would prove useful for future studies of the transcriptome of rhizomes at the critical developmental stage of rhizome bud formation.

Several additional developments in this study were apparent, including that segregation distortion occurred at all loci examined. This distortion was towards the non-rhizomatous parental allele. One possible explanation for this is that the adapted nature of the non-rhizomatous parent created secondary selection. Considering the proximity of the grain shattering allele (Lin et al. 2012) to our region of interest (Fig. 1), this is a very likely explanation. Linkage disequilibrium within this chromosome and our area of interest has been previously reported (Chittenden et al. 1994). As rhizome formation and non-shattering are linked in repulsion, this linkage must be overcome for successful integration of this QTL into grain sorghum cultivars. Another observation that is critical to introgressing this trait into elite sorghum cultivars is the amount of dominance effect from our QTL. The two over-wintering QTL in particular showed very small additive effects and strong dominance effects (Tables 3, 4). This suggests that these QTL have a heterozygous advantage. Because we used F4 individuals rather than the earlier generations used in previous studies (Paterson et al. 1995), we should have had less power for estimating dominance effects, but more for additive effects, making our discovery of dominance effects in this population even more meaningful. These results indicate that although an inbred rhizomatous over-wintering sorghum plant may be possible, using these inbreds in hybrid breeding schemes may produce an even more robust perennial plant.

## Conclusion

Two new QTL for over-wintering were identified. The targeted rhizome QTL interval was reduced by 75 %, from an area of approximately 58.7 Mb to an area of 14.5 Mb (calculating from the 2-LOD threshold of the 2010 data only). The number of candidate genes within the QTL region was similarly reduced by 53 %, from approximately 2,970–1,407. Furthermore, our phenotypic models showed that in this population rhizomes alone are not responsible for over-wintering, but are necessary. While more research is needed to identify the gene(s) and causal mechanisms responsible for over-wintering in sorghum, this study indicates that rhizomes are vital to over-wintering ability within our sorghum population. We suggest that research in this area continues, using more exact and accurate ways of measuring rhizome growth, larger populations, and higher density single nucleotide polymorphism genotyping, sequence-based genotyping, and/or transcriptome analysis.

The results of this study should aid in the creation of perennial sorghums that can over-winter in climates where they previously could not. These over-wintering sorghum types could be used for improvements in grain, forage, and biofuel sorghum production. They may prove particularly important because of their abilities to limit soil erosion, increase water use efficiency, and decrease fertilizer input needs. The results of this study may also be applicable to other crop species, both closely and distantly related.

## Electronic supplementary material

Below is the link to the electronic supplementary material.

Fig. S1 Daily maximum and minimum temperatures in College Station, Tx from November to March of the years 2010-2011 and 2011-2012. Temperatures are overlaid with normals for the same months calculated using data from 1981-2010 (OSC 2012) (TIFF 222 kb)
