# Supplementary figures and images for: Targeted mapping of quantitative trait locus regions for rhizomatousness in chromosome SBI-01 and analysis of overwintering in a Sorghum bicolor × S. propinquum population
Source: Mol Breed. 2012 Sep 6;31(1):153–62. doi: 10.1007/s11032-012-9778-8 (PMC3538016; doi:10.1007/s11032-012-9778-8)

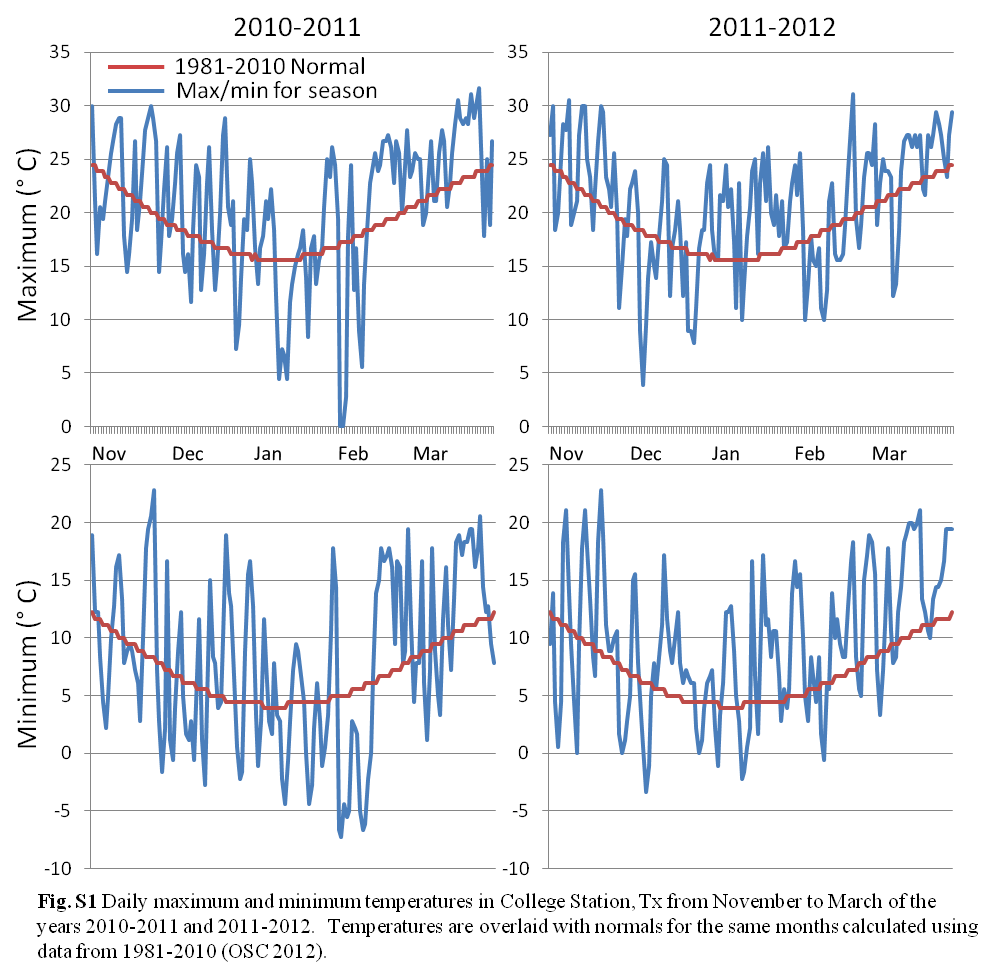

Supplement: Supplementary file 1 — Daily maximum and minimum temperatures in College Station, Tx from November to March of the years 2010-2011 and 2011-2012. Temperatures are overlaid with normals for the same months calculated using data from 1981-2010 (OSC 2012) (TIFF 222 kb) [file 11032_2012_9778_MOESM1_ESM.tif]
